# Vector competence of *Aedes aegypti* in transmitting Chikungunya virus: effects and implications of extrinsic incubation temperature on dissemination and infection rates

**DOI:** 10.1186/s12985-016-0566-7

**Published:** 2016-06-29

**Authors:** Sophiah Mbaika, Joel Lutomiah, Edith Chepkorir, Francis Mulwa, Christopher Khayeka-Wandabwa, Caroline Tigoi, Elijah Oyoo-Okoth, James Mutisya, Zipporah Ng’ang’a, Rosemary Sang

**Affiliations:** Institute of Tropical Medicine and Infectious Diseases (ITROMID), Jomo Kenyatta University of Agriculture and Technology (JKUAT), P.O. Box 62000–00200, Nairobi, Kenya; Centre for Virus Research (CVR), Kenya Medical Research Institute (KEMRI), P.O. Box 54628-00200, Nairobi, Kenya; International Centre of Insect Physiology and Ecology (ICIPE), P.O. Box 30772-00100, Nairobi, Kenya; African Population and Health Research Center (APHRC), P.O. Box 10787-00100, Nairobi, Kenya; Department of Natural Resource, School Natural Resources and Environmental Studies, Karatina University, P.O Box 1957-10101 Karatina, Kenya

**Keywords:** Infection rates, Dissemination rates, *Aedes aegypti*, Chikungunya virus, Extrinsic incubation temperature, Extrinsic incubation period

## Abstract

**Background:**

*Aedes aegypti* is a competent arthropod vector of chikungunya virus (CHIKV). The rate at which the virus disseminate in the vector is limited by temperature of their environment which can be an important determinant of geographical and seasonal limits to transmission by the arthropods in the tropics. This study investigated the vector competence of *Ae. aegypti* for CHIKV at ambient temperature of 32 and 26 °C (Coastal and Western Kenya respectively) reared at Extrinsic Incubation Temperature (EIT) of 32 and 26 °C that resembles those in the two regions.

**Methods:**

*Ae. aegypti* eggs were collected from coastal and Western Kenya, hatched in the insectary and reared to F_1_ generation. Four-day old mosquitoes were exposed to CHIKV through a membrane feeding. They were then incubated in temperatures mimicking the mean annual temperatures for Trans-Nzoia (26 °C) and Lamu (32 °C). After every 7, 10 and 13 days post infection (DPI); one third of exposed mosquitoes were sampled and assayed for virus infection and dissemination.

**Results:**

The midgut infection rates (MIR) of *Ae. aegypti* sampled from Coastal Region was significantly (*p* < 0.05) higher than those sampled from Western Kenya, with no statistical differences observed for the coastal *Ae. aegypti* at EIT 26 and at 32 °C. The MIR of *Ae. aegypti* from the Western Region was significantly (*p* < 0.05) affected by the EIT, with mosquito reared at EIT 32 °C exhibiting higher MIR than those reared at EIT 26 °C. There was a significant (*p* < 0.05) interactive effects of the region, EIT and DPI on MIR. The disseminated infection rates for the CHIKV in *Ae. aegypti* in the legs (DIR-L) was higher in mosquitoes sampled from Coast regardless of the EIT while those from Western Kenya, dissemination rates were significantly higher at higher EIT of 32 °C.

**Conclusions:**

Vector competence was higher in mosquito populations reared under high temperatures which weakens the midgut infection barrier. Hence, suggesting Lamu population is more susceptible to CHIKV therefore having a weaker mid gut infection barrier than the Trans Nzoia population. These underscores importance of examining the course of infection at various ambient temperatures and EIT between regions mosquito populations.

**Electronic supplementary material:**

The online version of this article (doi:10.1186/s12985-016-0566-7) contains supplementary material, which is available to authorized users.

## Background

Chikungunya fever is a self-remitting febrile viral illness caused by Chikungunya virus (CHIKV). The CHIKV is an arthropod-borne virus (arbovirus) of alphavirus genus in the family Togaviridae. The term “Chikungunya” was derived from the African dialect Swahili or Makonde and translates as “to be bent over and refers to the “stooped-over posture” exhibited by individuals with the disease [1]. The roots of this viral illness date back to 1953, when it was first detected in a Makonde Village in the Newala District of Tanzania [2, 3]. CHIKV infection is usually characterized by an acute onset of fever, rash, and arthralgias, and is often accompanied by headache, joint swelling and conjunctivitis [4–8]. Chikungunya disease is rarely fatal but is associated with significant morbidity. Although frequent outbreaks have been reported in the tropical countries of Africa and Southeast Asia, there are recent concern in Western countries and temperate zones around the world [9, 10]. In Africa, high prevalence of the CHIKV has been reported with first case being isolated in Tanzania in 1953 [1, 2], Union of the Comoros in 2005 [11], Congo (DRC) during 1998–2000 [12, 13], Central African Republic in 1999–2000 [14] and Mauritius and Madagascar in 2005 and 2006 respectively [15]. The dynamics attest to overall varying outbreak trends being observed in East/South/Central Africa and western Africa countries [11]. Kenya has experienced two outbreaks of chikungunya fever in 2004 [16] with the latest outbreak occurring in May 2016 in Northern Kenya (see Additional file 1) due to close proximity of mosquito breeding sites to human habitation and heavy rainfall [17]. Large variations in prevalence within these countries have also been reported such as the 59 % seroprevalence of the CHIKV infection in Busia District and 24 % in Malindi in Kenya [18].

The vectors principally responsible for transmission of the virus are *Aedes* mosquitoes [19, 20] where the virus actively replicates but the viral transmission occurs through the mosquitoes involved if the virus overcomes a series of anatomical barriers, i.e. the midgut and the salivary glands. In the past, large epidemics were related to the presence of the primary vector *Ae. aegypti*, which is also the main vector of the dengue virus [6, 21, 22]. *Ae. aegypti* was established in southern parts of continental Europe until the mid-1900s but subsequently disappeared for reasons that are yet to be completely understood [21]. In Africa, CHIKV apparently is maintained in a sylvatic transmission cycle involving primates and forest-dwelling *Aedes* mosquitoes [23]. Sylvatic vectors that have been implicated in transmission include *Ae. africanus* and *Ae. aegypti* in East Africa [24, 25]. *Ae. aegypti* predominantly breeds in stored fresh water, such as desert coolers, flower vases, water-tanks, etc., and in peri-domestic areas (discarded household junk items like vehicular tyres, coconut shells, pots, cans, bins, etc.) in urban and semi urban environments [26, 27]. Adult mosquitoes rest in cool and shady areas and bite humans during the daytime.

In mosquito infected by CHIKV, the extrinsic incubation period (EIP), the time from initial acquisition of pathogens until transmission is possible [28, 29], ranges from 2 to 9 days, with an average of 3 days [30, 31]. CHIKV is transmitted by *Aedes* mosquitoes, mainly by *Ae. aegypti.* The *Ae. aegypti*, is well distributed and is highly anthropophilic [32–34], thus increases the risk of CHIKV transmission. Mosquito vectors display different degrees of vector competence for different CHIKV isolates [35]. However, the invasive species *Ae. albopictus* has played a major role in most of recent epidemics since its last emergence in Kenya in 2004 [34, 36, 37]. Furthermore, recent studies have shown that transmission and spread of CHIKV in Africa and Asia is related to the CHIKV phylogroup and mosquito species [11, 14, 38, 39]. In the present study, CHIKV strain isolated from the 2004–2005 outbreak in Lamu Island was considered, the East/South/Central Africa and Indian Ocean genotype [14, 38]. Although the establishment of an arbovirus infection in a mosquito following ingestion of a virus is dependent on the amount of viral particles ingested by the mosquito and the susceptibility of the mosquito to infection by the virus [40], the vector competence is a complex trait involving an interplay between vectors, pathogens and environmental factors [35, 41, 42]. Temperature is regarded as one of the most important abiotic environmental factors affecting biological processes of mosquitoes, including interactions with arboviruses. Seasonal and geographic differences in temperature and anticipated climate change undoubtedly influence mosquito population dynamics, individuals’ traits related to vector biology (lifespan and vector competence for arboviruses), and disease transmission patterns. Extrinsic incubation temperature (EIT) has been shown to influence the replication and dissemination of arboviruses in vectors [43] thus altering the Extrinsic Incubation Period (EIP) [28, 29]. In the tropics, areas of high prevalence of the mosquitoes, with reported occurrence of CHIKV have variable temperature ranging between 25 and 34 °C throughout the year as part of climate characteristics. Yet information on the vector transmission of different populations of this species for CHIKV at different EIT is limited. Therefore the aim of this study was to compare the vector competence of coastal and Western Kenya *Ae. aegypti* populations for CHIKV under varying EIT. The coastal and western regions of Kenya have mean annual ambient temperature of 32 and 26 °C respectively. The information generated from this study provides data on competence factors that would influence epidemiological patterns of chikungunya fever.

## Methods

### Study setting

This study was conducted in Western Kenya and at the Coast. In Western Kenya, samples were obtained from Kiminini and Sasuri village in Trans Nzoia County while Lamu and Shela Village in Lamu County represented the Coastal region. Trans Nzoia has a latitude of 1.0567° N, and a longitude of 34.9507° E and a temperature range between 10 and 27 °C with an annual precipitation ranging between 1000 and 1200 mm, with the wettest months being experienced between April and October. The elevation of Kitale is about 1900 m. Meanwhile, Lamu County is situated in Kenya’s former Coast Province, at a longitude of 040°S´E and a latitude of 02°17´S, and is headquartered in Lamu town. The county covers a strip of northeastern coastal mainland and the Lamu Archipelago. It has a population of 101,539, and its land area is 6,167 km^2^. Lamu has a tropical savannah climate with average annual temperatures ranging between 13.6 and 40.9 °C. The county receives annual precipitation ranging between 900 and 1100 mm, with the rainy season experienced between May and June. From July the environment gradually gets hotter and dryer until March/April when it reaches 40 °C. Shela Village on Lamu Island is a tangle of narrow sandy lanes some smaller thatched dwellings and mosques. It is about 3.2 km south of Lamu Town. An additional file shows Map of the study areas in more detail (see Additional file 2).

### Mosquito eggs collection

Eggs of *Ae. aegypti* were collected using oviposition traps that consisted of black plastic cups lined with oviposition paper and half-filled with water. A total of 25 ovitraps were placed at least 50 m apart in each of the study setting. These ovitraps with oviposition papers were left in the peridomestic areas for four days. On the 4th day, all the ovicups were collected and the eggs transported to the biosafety level-2 (BSL-2) laboratory where they were dried on damp cotton wool to quiescent state as earlier described [44, 45] and stored in an air tight container at room temperature in the insectary.

### Mosquito rearing

Mosquitoes were reared in the insectary, maintained at a temperature of 28 °C and 80 % relative humidity (RH), with a 12:12-h (Light:Dark) photoperiod. The eggs were hatched and the larvae fed on TetraMin® fish food until pupation. Pupae were transferred to small plastic cups half-filled with water, placed in 4-l plastic cages screened with netting material on top and allowed to emerge into F_0_ adult mosquitoes*.* The emerging adults were then morphologically identified under a dissecting microscope using taxonomic keys of Edwards [46], to confirm that only *Ae. aegypti* mosquitoes were used in the subsequent experiments*.* The identified female *Ae. aegypti* were returned to experimental cages, blood fed on clean laboratory-bred mice and provided with oviposition papers to lay F_1_ eggs. The F_1_ eggs were hatched and reared as outlined and only adult female mosquitoes were used in the succeeding experiments [47].

### Preparation of virus stock

CHIKV strain isolated from the 2004–2005 outbreak in Lamu Island (Lamu001) was used in this study, the East/South/Central Africa and Indian Ocean genotype (group III), subgroup: East/South/Central African subgroup (IIIa and b) [14, 38]. The working stock virus was prepared by propagating CHIKV in Vero cells in T25 culture flasks. The infected cells were incubated at 37 °C and 5 % CO_2_ and observed daily for cytopathic changes before the virus was harvested. The virus stock titer was determined [log10 7.2 plaque-forming units (pfu)/mL] by plaque assay and aliquoted in cryovials and stored at −80 °C until usage [48].

### Oral infection of mosquitoes with the virus

Infection was performed in BSL2 insectary using four-day old mosquitoes after they were deprived of sucrose solution and water 24 h prior to exposure to the infectious blood meal using a membrane feeding apparatus [49]. A Hemotek membrane-feeding system (Discovery Workshops, Accrington, U.K.) which employed an electric heating element to maintain the temperature of the blood meal constant at 37 °C, was used. The infectious blood meal (log10 5.9 pfu/mL) was prepared by mixing 500 μl of the working virus with 500 μl of defribrinated sheep blood. The blood-virus mixture was pipetted into each membrane unit which was screwed onto the heating chamber and placed on top of mosquito cages each containing between 50–350 mosquitoes which were allowed to feed for 45 min [50–52]. Fully engorged mosquitoes were aspirated and transferred to empty 4-l plastic cages and maintained on 10 % glucose solution. These mosquitoes were reared for up to 13 days at low and high EITs of 26 °C or 32 °C respectively, mimicking the mean annual temperatures for Trans-Nzoia and Lamu counties respectively.

### Test for infection and dissemination rates of CHIKV

#### Mosquito sampling and dissections

A third of the fed mosquitoes were randomly sampled on day 7, 10, 13 post exposure (pe) and were dissected into abdomen, head and legs [25, 28, 53–55]. The abdomens of each mosquito were individually triturated in 1 mL of Eagle’s Minimum Essential Medium (MEM) (Sigma Aldrich) diluents and frozen at −80 °C until assayed for CHIKV by plaque assay on Vero cell monolayers. The dilutions were inoculated on confluent Vero cell monolayers in 12-well plates. Each plate was labeled with sample and dilution descriptions and for each virus stock serial dilutions (10^0^, 10^−1^, 10^−2^, 10^−3^, and 10^−4^) were made.

#### Virus assay in mosquito bodies

To demonstrate virus infection, 10 fold serial dilutions of the abdominal homogenates were made in Minimum Essential Medium Eagle (MEM), with Earle’s salts and reduced NaHCO_3_ (Sigma- Aldrich, St. Louis, MO) supplemented with 15 % FBS (Sigma-Aldrich), 2 % L-glutamine (Sigma- Aldrich) and 2 % antibiotic/antimycotic solution (Sigma-Aldrich) with 10,000 μl penicillin, 10 mg streptomycin and 25 μg amphotericin B per milliliter and tested for the presence of virus on Vero cell monolayers, by plaque assay, in 12-well plates. One hundred microliters of the appropriate dilutions of the abdominal homogenates was added to each of ten wells of the 12-well plate to infect the cells and the remaining two wells were used for controls. This was repeated to all the corresponding plates and dilutions. The plates were incubated at 37 °C and gently rocked every 15 min for 1 h to allow for virus adsorption. One milliliter of methyl cellulose solution (1.25 % Methylcellulose in MEM with 2 % FBS) was added gently to each well, and the plates incubated at 37 °C in a humidified 5 % CO_2_ incubator for 3 days. On the 3rd day, methylcellulose was removed from the wells using disposable plastic pipette in bio-safety cabinet. The plates were fixed by adding 1 ml of 10 % formaldehyde in PBS (1/10 diluted Formalin with PBS) over night and stained using 0.5 % crystal violet (0.5 g of powder form Crystal Violet in 100 mL of 99.5 % Ethanol) and after 1 h they were washed gently with tap water and left to dry at room temperature.

For the CHIKV positive abdomens, the corresponding legs and heads were homogenized and assayed as described to determine the dissemination status of the mosquitoes. Detection of the virus in the mosquito abdomen and not its legs or head was an indication that the mosquito had a non-disseminated infection (limited to its midgut), while detection of the virus in the legs and/or head was an indication of a disseminated infection [56–58].

### Ethical considerations

KEMRI Animal Care and Use Committee (ACUC), Scientific Steering Committee (SSC) and Ethical Review Committee (ERC) approved all the procedures used in this study. The guidelines were strictly adhered to during the research.

### Statistical analyses

The collected data were analyzed using SPSS version 20.0 software package. Differences in mosquito counts due to differences in treatments were analyzed using chi-square test. A logit model was utilized in showing the nominal main effects of the region, temperature, EIP and their interactions on the infection rates of CHIKV. MIR was computed as (Number of positive midgut infections divided by Total number of mosquitoes tested) × 100 % [59, 60] for day 7, 10 and 13 in the order. We defined the midgut infection rate as the percentage of mosquitoes tested that contained the virus in their abdomen/midgut and the dissemination rates in the legs and heads as the percentage of infected (abdomen/midgut positive) mosquitoes that contained virus in their legs and/or heads respectively. The differences between these groups were deemed statistically significant at *p* < 0.05.

## Results

### Midgut infection rates for Coastal and Western Kenya *Ae. aegypti* population

**Fig. 1:**
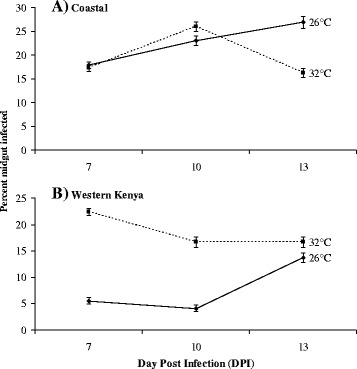
Proportion ± 95 % confidence interval of Coastal (**a**) and Western Kenya (**b**) *Ae. aegypti* infected at day 7, 10 and 13 post-infection at temperature levels of 26 and 32 °C

The 7, 10 and 13 days post infection (DPI) midgut infection rates of *Ae. aegypti* population for CHIKV in Coastal and Western Kenya *Ae. aegypti* reared at EIT of 26 and 32 °C is shown in Table 1 with temporal midgut infection rate vis-a-vis temperature ranges trends shown in Fig. 1. A binary logistic regression model showing the nominal effects of the region, EIT, DPI and their interactions on the Midgut infection rates (MIR) for CHIKV is presented in Table 2 (Model summary: −2 Log likelihood = 458.345, Nagelkerke R^2^ = 0.5323). Regardless of the EIT, the MIR of *Ae. aegypti* sampled from Coastal Region was significantly (*p* < 0.05) higher than those sampled from Western Kenya, with no statistical differences observed for the coastal *Ae. aegypti* at EIT 26 and at 32 °C. Meanwhile the MIR of *Ae. aegypti* from the Western Kenya Region (26 °C) was significantly (*p* < 0.05) affected by the EIT, with mosquito reared at EIT 32 °C exhibiting higher MIR than those reared at EIT 26 °C. The 2 way interactions between region, EIT and DPI significantly affected the MIR (*p* < 0.05). We also established a significant (*p* < 0.05) interactive effects of the region, EIT and DPI on MIR.

**Table 1:** Midgut infection rates of 7, 10 and 13 DPI of CHIKV in Coastal and Western Kenya *Ae. aegypti* reared at EIT of 26 and 32 °C

Region	Number midgut tested (n)	EIT^a^	DPI^b^	Number of midgut infections	MIR^c^ (%)
Coastal	156	26 °C	7		17.9
			10		23.1
			13		26.9
	173	32 °C	7		17.3
			10		26.0
			13		16.2
Western Kenya	146	26 °C	7		5.5
			10		4.1
			13		13.7
	156	32 °C	7		22.4
			10		16.7
			13		16.7

Within each population and temperature conditions, the number of tested mosquitoes is the same for all the days of sampling

^a^EIT *=* Extrinsic Incubation Temperature

^b^DPI *=* Days Post Infection

^c^MIR *=* Midgut Infection Rate

**Table 2:** Logit model of the nominal main effects of the region, EIT and DPI and their interactions on the midgut infection rates of CHIKV

Effects	E	Std Error	df	Wald	Sig	Chi-square	EXP(B)
Intercept	−0.742	4.7348	1	6.8119	0.0037	5.9431	0.476
Region	1.5207	5.2578	1	10.1012	0.0000	37.6654	4.5754
EIT	1.039	3.6764	1	−6.3544	0.0021	17.8042	2.827
DPI	−0.602	4.3498	1	7.3677	0.0027	6.1545	0.548
Region*EIT	0.459	3.1241	2	8.4674	0.0004	9.1973	1.5825
Region*DPI	1.518	9.3498	2	−19.2382	0.0000	31.4455	4.563
EIT *DPI	0.690	6.0174	2	7.01305	0.0002	21.1073	1.994
Region* EIT *DPI	−0.6027	2.1132	3	0.0065	0.0331	4.3053	0.5473

EIT = Extrinsic Incubation Temperature, DPI = Days Post Infection

### Dissemination rates of CHIKV in Coastal and Western Kenya *Ae. aegypti* population

The dissemination rates of infected *Ae. aegypti* for CHIKV from the Coastal Region and Western Kenya at 7, 10 and 13 DPI and at EIT of 26 and 32 °C is shown in Table 3. The disseminated infection rates for the CHIKV in *Ae. aegypti* in the legs (DIR-L) was higher in mosquito sampled from higher ambient temperature setting (Coastal Region) regardless of the EIT while those from ambient temperature of 26 °C (Western Kenya) dissemination rates was significantly higher at higher EIT of 32 °C. There were no significant interactions (*p* > 0.05) between region and EIT on the dissemination of CHIKV from the midgut to the head, indicating that the trends in the infection rates by CHIKV remained similar at the coastal and Western region regardless of the EIT. Notably, there were no significant (*p* > 0.05) interaction between region, EIT and DPI on DIR on the legs and head.

**Table 3:** Dissemination rates of CHIKV to the legs and head in Coastal and Western Kenya *Ae. aegypti* reared at Extrinsic Incubation Temperature of 26 and 32 °C

				Number infected^(b)^		Dissemination rates (%) ^(c)^	
Region	Number of midgut infections ^(a)^	EIT	DPI	Legs	Head	Legs	Head
Coastal Region	106	26 °C	7	19	11	17.9	10.4
			10	27	3	25.5	2.8
			13	18	3	17.0	2.8
	103	32 °C	7	7	3	6.8	2.9
			10	21	17	20.4	16.5
			13	30	27	29.1	26.2
Western Kenya	34	26 °C	7	9	9	26.5	26.5
			10	4	6	11.8	17.6
			13	7	7	20.6	20.6
	87	32 °C	7	25	22	28.7	25.3
			10	15	7	17.2	8.0
			13	23	20	26.4	23.0

Dissemination rate (**c**) for legs or head in that order is provided as [(**b** dived by **a**) × 100 %] where numerator **b** corresponds to number of infected legs or head respectively

## Discussion

Arboviruses are ecologically complex, and interactions between larval mosquitoes and their aquatic environment can influence adult transmission dynamics. Moreover, due to the impact of climate on vector ecology, competence and their risk of transmitting viruses may be sensitive to projected changes in global temperatures. In this study, we evaluated the effect of ambient temperatures and changes of EIT on the risk of vector transmission and competence of the *Ae. aegypti* for CHIKV. We provide evidence that the incubation temperatures of vector directly impact virus transmission by influencing the likelihood of infection and dissemination of CHIKV. We established that the MIR of *Ae. aegypti* sampled from the coastal area with ambient temperature of 32 °C was higher than those sampled from the western Kenya that has ambient temperature 26 °C regardless of the EIT. Meanwhile for *Ae. aegypti* emanating from lower ambient temperature of 26 °C, there was increased MIR when EIT was increased from 26 to 32 °C. This suggests that virus transmission is likely to be affected more by higher environmental temperature due to possible effects of the temperature on the biological processes moderating the vector competence [61]. It has earlier been noted that temperature may limit virus transmission in areas where the vectors is present noting that an increase in environmental temperature for adult mosquitoes reduces the EIP most likely due to an increase in the metabolism of the adult mosquito and replication speed of the virus [28, 61, 62]. Equally, temperature changes experienced in the immature stages of the mosquito development before infection may affect vector virus interactions by changing physical and physiological characteristics of mid-gut barriers which would impact virus infection and transmission [63, 64]. This is in agreement with previous studies which have established that ambient temperature affect the biological processes of mosquitoes and plays a key role in modulating mosquito vector competence for pathogens [65–67]. Previous studies have indicated that increases in adult-holding incubation temperatures have usually been associated with enhanced vector competence [62, 68–74]. However, some studies have identified reduced vector competence and activity in nature associated with increases in incubation temperature [64, 75–77]. It has long been recognized that increases in incubation temperature reduce the extrinsic incubation period (the time from initial acquisition of pathogens until transmission is possible) [28], which render virus transmission more likely under such incubation period. Along the same lines, increases in temperature reduce the adult lifespan of mosquitoes and may impinge transmission [69, 70, 78]. Temperature effects may drastically alter risk of disease transmission, especially under conditions where the extrinsic incubation period approaches the lifespan of the mosquito. This result differs with other systems where arboviral vector competence was reduced in female mosquitoes that were reared at higher compared to lower temperatures [68–70, 78]. However, vector capacity of a mosquito population is a complex phenomenon that is influenced by a number of factors such as host seeking behavior and longevity of the infected mosquitoes apart from temperature and inherent factors [79] and thus further studies are recommended on how these factors can combine to affect the MIR.

Mosquito susceptibility to arbovirus infection resides primarily in the midgut and can vary greatly between mosquito species and geographical strains of the same species and even within individuals of the same population [80]. Vector competence, which is the capacity of an arthropod to acquire an infection and transmit it to a subsequent host, can greatly vary among individuals and between populations [56] and has been previously linked to genetics [81] as well as by climate variables such as temperature [70, 82]. Disseminated infection is generally accepted as a measure of a mosquito’s ability to transmit a virus through biting [56, 70]. The rate of dissemination, when expressed as a percentage of the number of mosquitoes infected, may provide information about the effect of a “midgut escape barrier” moderating whether gut infections are able to disseminate into the hemolymph. In the current study, the dissemination rates of infected *Ae. aegypti* for CHIKV in the legs was high at higher ambient temperature regardless of the EIT. Notably, the disseminated infection rates for the CHKV in *Ae. aegypti* in the legs was higher in mosquito emanating from ambient temperature 32 °C (Coastal Region) regardless of the EIT while those from ambient temperature of 26 °C (Western Kenya) dissemination rates was significantly higher at higher EIT of 32 °C. These results suggest that the midgut barriers preventing dissemination were strongly influenced by the ambient and rearing temperature. Thus, it can be speculated that there may be an increased midgut escape barrier in mosquitoes derived from the higher rearing temperatures. At temperature of 26 °C during the adult stage resulted in the lowest rates of viral dissemination. Rates of dissemination were higher at 32 °C relative to cooler holding temperatures of adults. These results corroborate observations found for laboratory studies examining susceptibility to dengue virus infection and length of the extrinsic incubation period in *Ae. albopictus* and *Ae. aegypti* [83–85]. However, we found no association between vector dissemination in between the midgut and the head. The explanation for these observed effects of mosquitoes with disseminated infections is not entirely clear, but it does suggest complex effects of temperature on virus infection and dissemination and by extension, mosquito competence.

## Conclusion

The current study underscores the importance of the environmental and incubation temperature in dictating the vector epidemiological risk of the virus in the human populations. Vector competence was higher in mosquito populations reared under high temperatures which weakens the midgut infection barrier. Hence, suggesting Lamu population is more susceptible to CHIKV therefore having a weaker mid gut infection barrier than the Trans Nzoia population. This study further demonstrates the importance of examining the course of infection at various ambient temperatures and EIT between the two mosquito populations. Vector control measures should be triggered as an integral component of climate change policies discourse to prevent un-anticipated transmission transition of such vector ecology dependent infectious pathogens. Although our results show differences in vector competence, other factors (mosquito densities, feeding behavior, mosquito survival rates) composing the vector capacity, are needed to assess more accurately the risk of CHIKV transmission alongside virus titers in the context of dissemination. Future studies should explore the connection between larval rearing temperature-infection patterns observed in the laboratory and patterns in the field, and how climate and climate change related factors may continue to impact the mosquito larval environment and the epidemiology of CHIKV.

## Additional files

Additional file 1: Auxiliary materials on CHIKV epidemics in Kenya. (DOC 26 kb) Additional file 2: Map of the study areas. (DOC 1546 kb)

## References

[1] Ross R (1956). The Newala epidemic: III. The virus: isolation, pathogenic properties and relationship to the epidemic. J Hyg.

[2] Robinson MC (1955). An epidemic of virus disease in Southern Province, Tanganyika territory, in 1952–1953. Trans R Soc Trop Med Hyg.

[3] Lumsden W (1955). An epidemic of virus disease in Southern Province, Tanganyika territory, in 1952–1953 II. General description and epidemiology. Trans R Soc Trop Med Hyg.

[4] Gérardin P, Fianu A, Malvy D, Mussard C, Boussaïd K, Rollot O, Michault A, Gaüzere B-A, Bréart G, Favier F (2011). Perceived morbidity and community burden after a Chikungunya outbreak: the TELECHIK survey, a population-based cohort study. BMC Medicine.

[5] Moro M, Grilli E, Corvetta A, Silvi G, Angelini R, Mascella F, Miserocchi F, Sambo P, Finarelli A, Sambri V (2012). Long-term chikungunya infection clinical manifestations after an outbreak in Italy: a prognostic cohort study. J Infect.

[6] Pialoux G, Gaüzère B-A, Jauréguiberry S, Strobel M (2007). Chikungunya, an epidemic arbovirosis. Lancet Infect Dis.

[7] Nunes MR, Faria NR, de Vasconcelos JM, Golding N, Kraemer MU, de Oliveira LF, Azevedo RS, da Silva DE, da Silva EV, da Silva SP (2015). Emergence and potential for spread of Chikungunya virus in Brazil. BMC Medicine.

[8] Schilte C, Staikowsky F, Couderc T, Madec Y, Carpentier F, Kassab S, Albert ML, Lecuit M, Michault A (2013). Chikungunya virus-associated long-term arthralgia: a 36-month prospective longitudinal study. PLoS Negl Trop Dis.

[9] Staples JE, Breiman RF, Powers AM (2009). Chikungunya fever: an epidemiological review of a re-emerging infectious disease. Clin Infect Dis.

[10] Mohan A, Kiran D, Manohar IC, Kumar DP (2010). Epidemiology, clinical manifestations, and diagnosis of Chikungunya fever: lessons learned from the re-emerging epidemic. Indian J Dermatol.

[11] Powers AM, Logue CH (2007). Changing patterns of chikungunya virus: re-emergence of a zoonotic arbovirus. J Gen Virol.

[12] Pastorino B, Muyembe‐Tamfum J, Bessaud M, Tock F, Tolou H, Durand J, Peyrefitte C (2004). Epidemic resurgence of Chikungunya virus in democratic Republic of the Congo: identification of a new central African strain. J Med Virol.

[13] Nur YA, Groen J, Heuvelmans H, Tuynman W, Copra C, Osterhaus A (1999). An outbreak of West Nile fever among migrants in Kisangani, Democratic Republic of Congo. AmJTrop Med Hyg.

[14] Njenga MK, Nderitu L, Ledermann J, Ndirangu A, Logue C, Kelly C, Sang R, Sergon K, Breiman R, Powers A (2008). Tracking epidemic chikungunya virus into the Indian Ocean from East Africa. J Gen Virol.

[15] Ravi V (2006). Re-emergence of chikungunya virus in India. Indian J Med Microbiol.

[16] Sergon K, Njuguna C, Kalani R, Ofula V, Onyango C, Konongoi LS, Bedno S, Burke H, Dumilla AM, Konde J (2008). Seroprevalence of chikungunya virus (CHIKV) infection on Lamu Island, Kenya, October 2004. AmJTrop Med Hyg.

[17] Anyamba A, Small JL, Britch SC, Tucker CJ, Pak EW, Reynolds CA, Crutchfield J, Linthicum KJ (2014). Recent weather extremes and impacts on agricultural production and vector-borne disease outbreak patterns. PLoS One.

[18] Mease LE, Coldren RL, Musila LA, Prosser T, Ogolla F, Ofula VO, Schoepp RJ, Rossi CA, Adungo N (2011). Seroprevalence and distribution of arboviral infections among rural Kenyan adults: A cross-sectional study. Virol J.

[19] Jupp P, McIntosh B (1988). Chikungunya virus disease. The Arboviruses: Epidemiology and Ecology.

[20] Jupp P, McIntosh B (1990). Aedes furcifer and other mosquitoes as vectors of chikungunya virus at Mica, northeastern Transvaal, South Africa. J Am Mosq Control Assoc.

[21] Reiter P (2010). Yellow fever and dengue: a threat to Europe. Euro Surveill.

[22] Lambrechts L, Scott TW, Gubler DJ (2010). Consequences of the expanding global distribution of Aedes albopictus for dengue virus transmission. PLoS Negl Trop Dis.

[23] Vanlandingham DL, Hong C, Klingler K, Tsetsarkin K, McElroy KL, Powers AM, Lehane MJ, Higgs S (2005). Differential infectivities of o’nyong-nyong and chikungunya virus isolates in Anopheles gambiae and Aedes aegypti mosquitoes. AmJTrop Med Hyg.

[24] McCrae A, Henderson B, Kirya B, Sempala S (1971). Chikungunya virus in the Entebbe area of Uganda: isolations and epidemiology. Trans R Soc Trop Med Hyg.

[25] Diallo M, Thonnon J, Traore-Lamizana M, Fontenille D (1999). Vectors of Chikungunya virus in Senegal: current data and transmission cycles. AmJTrop Med Hyg.

[26] WHO, Research SPf, Diseases TiT, Diseases WHODoCoNT, Epidemic WHO, Alert P (2009). Dengue: guidelines for diagnosis, treatment, prevention and control.

[27] Samuel PP, Krishnamoorthi R, Hamzakoya K, Aggarwal C (2009). Entomo-epidemiological investigations on chikungunya outbreak in the Lakshadweep islands, Indian Ocean. Indian J Med Res.

[28] Chamberlain RW, Sudia WD (1955). The effects of temperature upon the extrinsion incubation of eastern equine encephalitis in mosquitoes. Am J Epidemiol.

[29] Patz JA, Epstein PR, Burke TA, Balbus JM (1996). Global climate change and emerging infectious diseases. JAMA.

[30] Scott TW, Burrage TG (1984). Rapid infection of salivary glands in Culiseta melanura with eastern equine encephalitis virus: an electron microscopic study. AmJTrop Med Hyg.

[31] Rudolph KE, Lessler J, Moloney RM, Kmush B, Cummings DA (2014). Incubation periods of mosquito-borne viral infections: a systematic review. AmJTrop Med Hyg.

[32] Harrington LC, Edman JD, Scott TW (2001). Why do female Aedes aegypti (Diptera: Culicidae) feed preferentially and frequently on human blood?. J Med Entomol.

[33] Sang RC, Ahmed O, Faye O, Kelly CL, Yahaya AA, Mmadi I, Toilibou A, Sergon K, Brown J, Agata N (2008). Entomologic investigations of a chikungunya virus epidemic in the Union of the Comoros, 2005. AmJTrop Med Hyg.

[34] Charrel RN, de Lamballerie X, Raoult D (2007). Chikungunya outbreaks-the globalization of vectorborne diseases. N Engl J Med.

[35] Zouache K, Fontaine A, Vega-Rua A, Mousson L, Thiberge J-M, Lourenco-De-Oliveira R, Caro V, Lambrechts L, Failloux A-B (2014). Three-way interactions between mosquito population, viral strain and temperature underlying chikungunya virus transmission potential. Proc R Soc Lond B Biol Sci.

[36] Talbalaghi A, Moutailler S, Vazeille M, FAILLOUX AB (2010). Are Aedes albopictus or other mosquito species from northern Italy competent to sustain new arboviral outbreaks?. Med Vet Entomol.

[37] Wikan N, Sakoonwatanyoo P, Ubol S, Yoksan S, Smith DR (2012). Chikungunya virus infection of cell lines: analysis of the East, Central and South African lineage. PLoS One.

[38] Cui J, Gao M, Ren X (2011). Phylogeny and homologous recombination in Chikungunya viruses. Infect Genet Evol.

[39] Volk SM, Chen R, Tsetsarkin KA, Adams AP, Garcia TI, Sall AA, Nasar F, Schuh AJ, Holmes EC, Higgs S (2010). Genome-scale phylogenetic analyses of chikungunya virus reveal independent emergences of recent epidemics and various evolutionary rates. J Virol.

[40] Clements A (1963). The physiology of mosquitoes. International series of monographs on pure and applied biology.

[41] McFarlane M, Arias-Goeta C, Martin E, O'Hara Z, Lulla A, Mousson L, Rainey SM, Misbah S, Schnettler E, Donald CL (2014). Characterization of Aedes aegypti innate-immune pathways that limit Chikungunya virus replication.

[42] Shang C-S, Fang C-T, Liu C-M, Wen T-H, Tsai K-H, King C-C (2010). The role of imported cases and favorable meteorological conditions in the onset of dengue epidemics. PLoS Negl Trop Dis.

[43] Organization WH (2007). Outbreak and spread of chikungunya. Wkly Epidemiol Rec.

[44] Steinly B, Novak R, Webb D (1991). A new method for monitoring mosquito oviposition in artificial and natural containers. J Am Mosq Control Assoc.

[45] Chepkorir E, Lutomiah J, Mutisya J, Mulwa F, Limbaso K, Orindi B, Sang R (2014). Vector competence of Aedes aegypti populations from Kilifi and Nairobi for dengue 2 virus and the influence of temperature. Parasites Vectors.

[46] Edwards FW (1941). Mosquitoes of the Ethiopian Region. III.-Culicine adults and pupae. Mosquitoes of the Ethiopian Region III-Culicine Adults and Pupae.

[47] Gerberg EJ, Barnard DR, Ward RA (1994). Manual for mosquito rearing and experimental techniques.

[48] Reiskind MH, Pesko K, Westbrook CJ, Mores CN (2008). Susceptibility of Florida mosquitoes to infection with chikungunya virus. AmJTrop Med Hyg.

[49] Novak M, Berry W, Rowley W (1991). Comparison of four membranes for artificially bloodfeeding mosquitoes. J Am Mosq Control Assoc.

[50] Cosgrove J, Wood R, Petrić D, Evans D, Abbott R (1994). A convenient mosquito membrane feeding system. J Am Mosq Control Assoc.

[51] Hagen H, Grunewald J (1990). Routine blood-feeding of Aedes aegypti via a new membrane. J Am Mosq Control Assoc.

[52] Foggie T, Achee N (2009). Standard operating procedures: rearing Aedes aegypti for the HITSS and Box laboratory assays. USUHS [Internet].

[53] Dupont-Rouzeyrol M, Caro V, Guillaumot L, Vazeille M, D'Ortenzio E, Thiberge J-M, Baroux N, Gourinat A-C, Grandadam M, Failloux A-B (2012). Chikungunya virus and the mosquito vector Aedes aegypti in New Caledonia (South Pacific Region). Vector-Borne Zoonotic Dis.

[54] Dubrulle M, Mousson L, Moutailler S, Vazeille M, Failloux A-B (2009). Chikungunya virus and Aedes mosquitoes: saliva is infectious as soon as two days after oral infection. PLoS One.

[55] Vazeille M, Moutailler S, Coudrier D, Rousseaux C, Khun H, Huerre M, Thiria J, Dehecq J-S, Fontenille D, Schuffenecker I (2007). Two Chikungunya isolates from the outbreak of La Reunion (Indian Ocean) exhibit different patterns of infection in the mosquito, Aedes albopictus. PloS One.

[56] Turell MJ, Beaman JR, Tammariello RF (1992). Susceptibility of selected strains of Aedes aegypti and Aedes albopictus (Diptera: Culicidae) to chikungunya virus. J Med Entomol.

[57] Mourya D, Thakare J, Gokhale M, Powers A, Hundekar S, Jayakumar P, Bondre V, Shouche Y, Padbidri V (2000). Isolation of chikungunya virus from Aedes aegypti mosquitoes collected in the town of Yawat, Pune District, Maharashtra State, India. Acta Virol.

[58] Myers R, Carey D, Reuben R, Jesudass E, De Ranitz C, Jadhav M (1965). The 1964 epidemic of dengue-like fever in South India: isolation of chikungunya virus from human sera and from mosquitoes. Indian J Med Res.

[59] Lutomiah JL, Koka H, Mutisya J, Yalwala S, Muthoni M, Makio A, Limbaso S, Musila L, Clark JW, Turell MJ (2011). Ability of selected Kenyan mosquito (Diptera: Culicidae) species to transmit West Nile virus under laboratory conditions. J Med Entomol.

[60] Turell MJ, Gargan T, Bailey CL (1984). Replication and dissemination of Rift Valley fever virus in Culex pipiens. AmJTrop Med Hyg.

[61] Purse BV, Mellor PS, Rogers DJ, Samuel AR, Mertens PP, Baylis M (2005). Climate change and the recent emergence of bluetongue in Europe. Nat Rev Microbiol.

[62] Davis NC (1932). The effect of various temperatures in modifying the extrinsic incubation period of the yellow fever virus in Aedes aegypti. Am J Epidemiol.

[63] Westbrook CJ, Reiskind MH, Pesko KN, Greene KE, Lounibos LP (2010). Larval environmental temperature and the susceptibility of Aedes albopictus Skuse (Diptera: Culicidae) to Chikungunya virus. Vector-Borne Zoonotic Dis.

[64] Hardy J, Meyer R, Presser S, Milby M (1990). Temporal variations in the susceptibility of a semi-isolated population of Culex tarsalis to peroral infection with western equine encephalomyelitis and St. Louis encephalitis viruses. AmJTrop Med Hyg.

[65] Lambrechts L, Paaijmans KP, Fansiri T, Carrington LB, Kramer LD, Thomas MB, Scott TW (2011). Impact of daily temperature fluctuations on dengue virus transmission by Aedes aegypti. Proc Natl Acad Sci.

[66] Murdock CC, Paaijmans KP, Cox-Foster D, Read AF, Thomas MB (2012). Rethinking vector immunology: the role of environmental temperature in shaping resistance. Nat Rev Microbiol.

[67] Lefevre T, Vantaux A, Dabire KR, Mouline K, Cohuet A (2013). Non-genetic determinants of mosquito competence for malaria parasites. PLoS Pathog.

[68] Kay BH, Fanning ID, Mottram P (1989). The vector competence of Culex annulirostris, Aedes sagax and Aedes alboannulatus for Murray Valley encephalitis virus at different temperatures. Med Vet Entomol.

[69] Kay BH, Fanning ID, Mottram P (1989). Rearing temperature influences flavivirus vector competence of mosquitoes. Med Vet Entomol.

[70] Turell MJ (1993). Effect of environmental temperature on the vector competence of Aedes taeniorhynchus for Rift Valley fever and Venezuelan equine encephalitis viruses. AmJTrop Med Hyg.

[71] Richards SL, Lord CC, Pesko K, Tabachnick WJ (2009). Environmental and biological factors influencing Culex pipiens quinquefasciatus Say (Diptera: Culicidae) vector competence for Saint Louis encephalitis virus. AmJTrop Med Hyg.

[72] Richards SL, Mores CN, Lord CC, Tabachnick WJ (2007). Impact of extrinsic incubation temperature and virus exposure on vector competence of Culex pipiens quinquefasciatus Say (Diptera: Culicidae) for West Nile virus. Vector-Borne Zoonotic Dis.

[73] Kilpatrick AM, Meola MA, Moudy RM, Kramer LD (2008). Temperature, viral genetics, and the transmission of West Nile virus by Culex pipiens mosquitoes.

[74] Anderson SL, Richards SL, Tabachnick WJ, Smartt CT (2010). Effects of West Nile virus dose and extrinsic incubation temperature on temporal progression of vector competence in Culex pipiens quinquefasciatus. J Am Mosq Control Assoc.

[75] Kramer LD, Hardy JL, Presser SB (1983). Effect of temperature of extrinsic incubation on the vector competence of Culex tarsalis for western equine encephalomyelitis virus. AmJTrop Med Hyg.

[76] Muturi EJ, Alto BW (2011). Larval environmental temperature and insecticide exposure alter Aedes aegypti competence for arboviruses. Vector-Borne Zoonotic Dis.

[77] Muturi EJ, Lampman R, Costanzo K, Alto BW (2011). Effect of temperature and insecticide stress on life-history traits of Culex restuans and Aedes albopictus (Diptera: Culicidae). J Med Entomol.

[78] Hardy JL, Rosen L, Kramer LD, Presser SB, Shroyer DA, Turell MJ (1980). Effect of rearing temperature on transovarial transmission of St. Louis encephalitis virus in mosquitoes. AmJTrop Med Hyg.

[79] Lutomiah J, Bast J, Clark J, Richardson J, Yalwala S, Oullo D, Mutisya J, Mulwa F, Musila L, Khamadi S (2013). Abundance, diversity, and distribution of mosquito vectors in selected ecological regions of Kenya: public health implications. J Vector Ecol.

[80] Hardy JL, Houk EJ, Kramer LD, Reeves WC (1983). Intrinsic factors affecting vector competence of mosquitoes for arboviruses. Annu Rev Entomol.

[81] Mercado-Curiel RF, Black WC, de L Muñoz M (2008). A dengue receptor as possible genetic marker of vector competence in Aedes aegypti. BMC Microbiol.

[82] Dohm DJ, O'Guinn ML, Turell MJ (2002). Effect of environmental temperature on the ability of Culex pipiens (Diptera: Culicidae) to transmit West Nile virus. J Med Entomol.

[83] Watts DM, Burke DS, Harrison BA, Whitmire RE, Nisalak A (1986). Effect of temperature on the vector efficiency of Aedes aegypti for dengue 2 virus. DTIC Document.

[84] Rohani A, Wong Y, Zamre I, Lee H, Zurainee M (2009). The effect of extrinsic incubation temperature on development of dengue serotype 2 and 4 viruses in Aedes aegypti (L.). Southeast Asian J Trop Med Public Health.

[85] Xiao F-Z, Zhang Y, Deng Y-Q, He S, Xie H-G, Zhou X-N, Yan Y-S (2014). The effect of temperature on the extrinsic incubation period and infection rate of dengue virus serotype 2 infection in Aedes albopictus. Arch Virol.

