# Supplementary material for: Vector competence of Aedes aegypti in transmitting Chikungunya virus: effects and implications of extrinsic incubation temperature on dissemination and infection rates
Source: Virol J. 2016 Jun 29;13:114. doi: 10.1186/s12985-016-0566-7 (PMC4928303; doi:10.1186/s12985-016-0566-7)
Supplement: Additional file 1: — Auxiliary materials on CHIKV epidemics in Kenya. (DOC 26 kb) [file 12985_2016_566_MOESM1_ESM.doc]

**Additional File 1: Auxiliary materials on CHIKV epidemics in Kenya**

<http://www.standardmedia.co.ke/health/article/2000203242/alarm-over-chikungunya-outbreak>

<http://www.the-star.co.ke/news/2016/05/26/state-dispatches-10-experts-to-mandera-to-tackle-chikungunya_c1358694>

<https://www.youtube.com/watch?v=0KCs9BoQq60>
