# Supplementary material for: Vector competence of Aedes aegypti in transmitting Chikungunya virus: effects and implications of extrinsic incubation temperature on dissemination and infection rates
Source: Virol J. 2016 Jun 29;13:114. doi: 10.1186/s12985-016-0566-7 (PMC4928303; doi:10.1186/s12985-016-0566-7)
Supplement: Additional file 2: — Map of the study areas. (DOC 1546 kb) [file 12985_2016_566_MOESM2_ESM.doc]

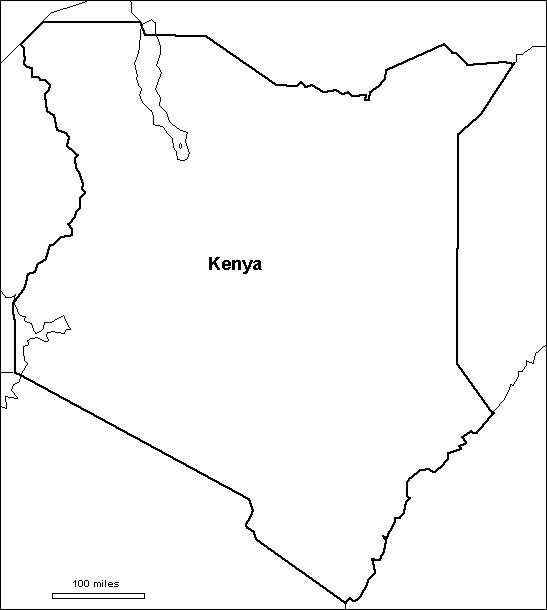


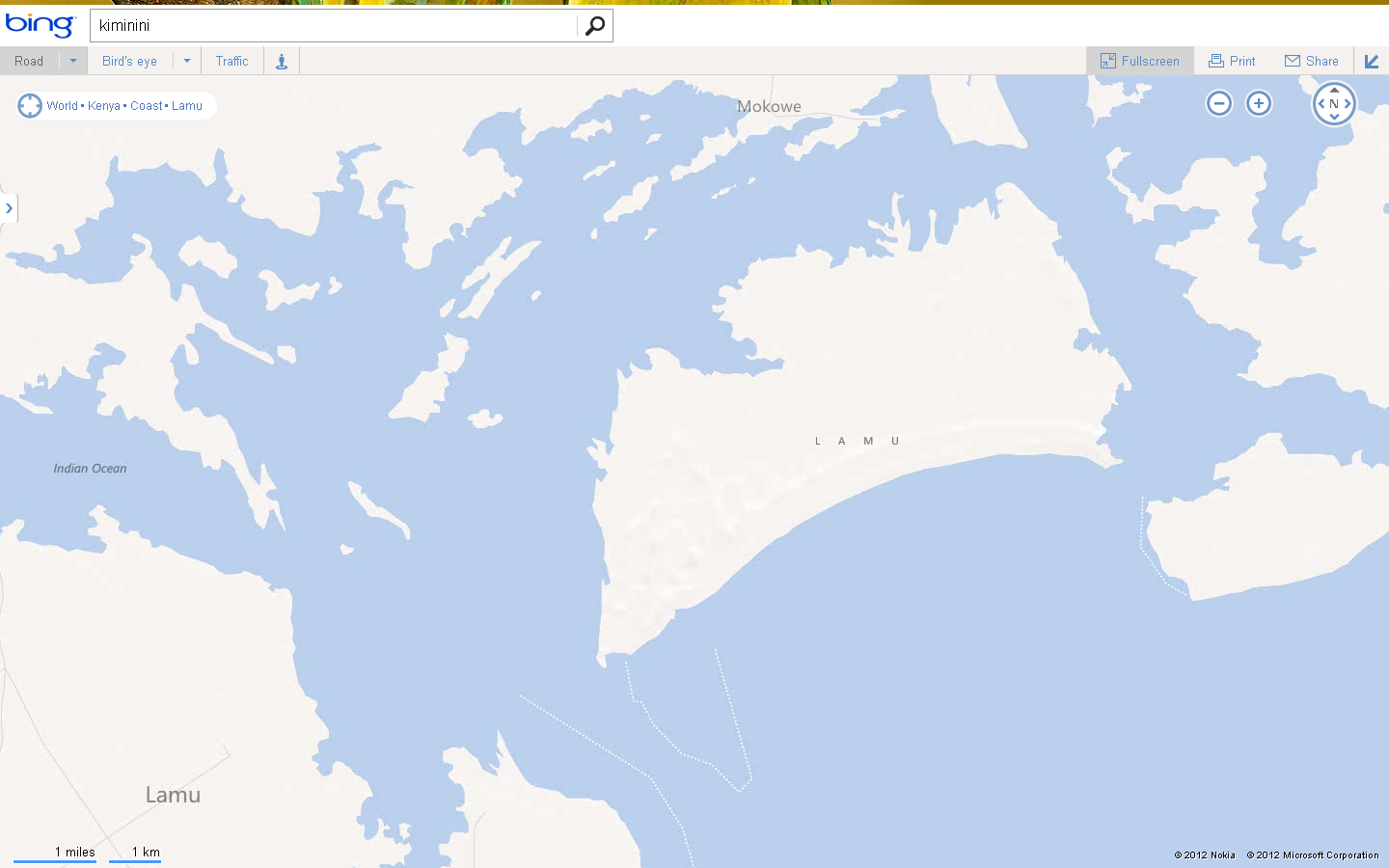


Lamu Market

Shela Village

Lamu Island

Lake Victoria

Lake Turkana

Indian Ocean


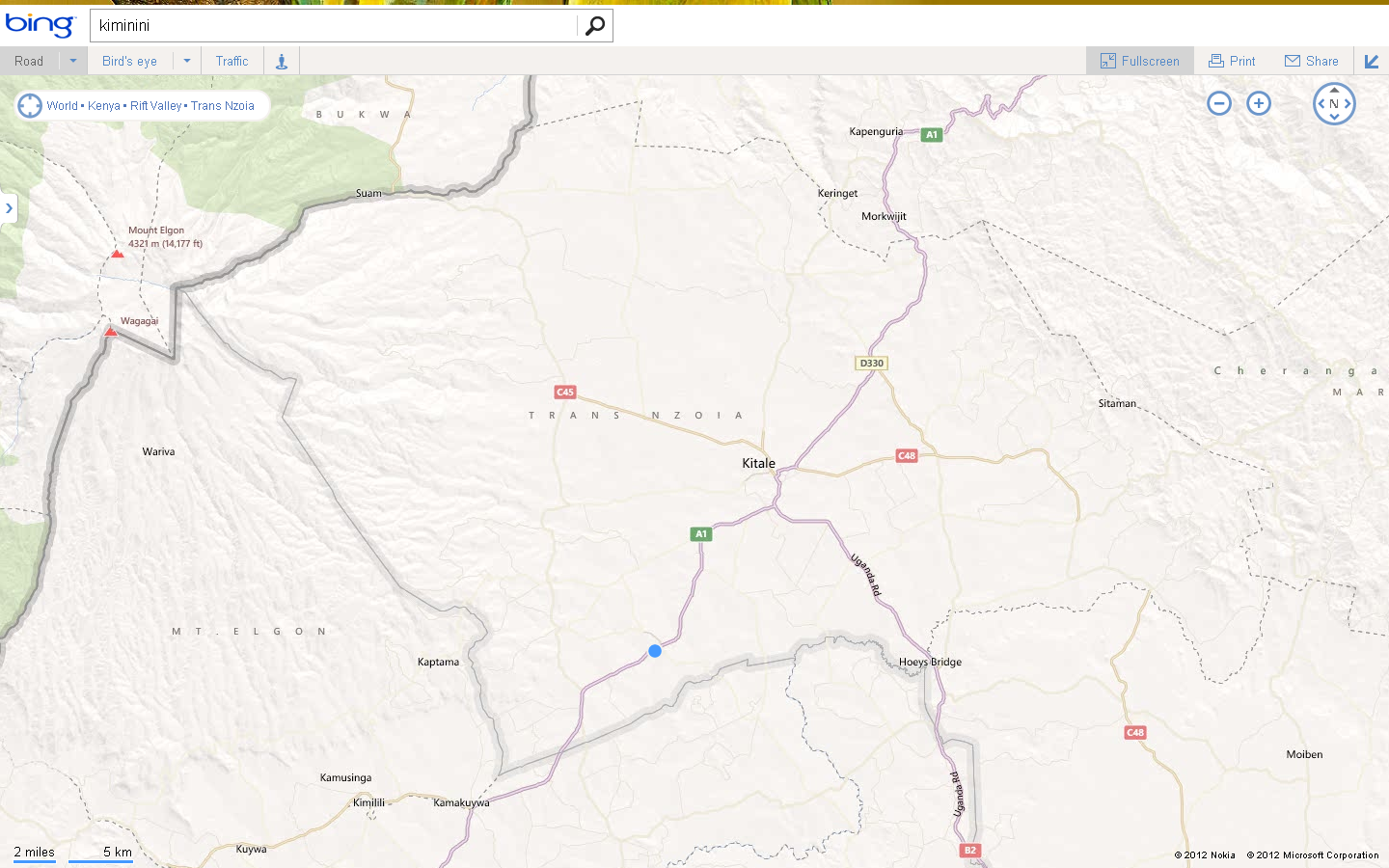


Kiminini Market

T**rans Nzoia**

Sasuri Village

Mt. Elgon Forest

Kiminini Market

**Additional File 2: Map of the study areas**
